# Physical Activity, Cardiorespiratory Fitness, and the Obesity Paradox with Consideration for Racial and/or Ethnic Differences: A Broad Review and Call to Action

**DOI:** 10.31083/j.rcm2508291

**Published:** 2024-08-19

**Authors:** Joshua R. Sparks, Xuewen Wang, Carl J. Lavie, Xuemei Sui

**Affiliations:** ^1^Department of Exercise Science, Norman J. Arnold School of Public Health, University of South Carolina, Columbia, SC 29208, USA; ^2^Expeditionary and Cognitive Sciences Research Group, Department of Warfighter Performance, Naval Health Research Center, Leidos Inc. (Contract), San Diego, CA 92106, USA; ^3^Department of Cardiovascular Disease, John Ochsner Heart and Vascular Institute, Ochsner Clinical School, University of Queensland School of Medicine, New Orleans, LA 70121, USA

**Keywords:** cardiorespiratory fitness, obesity, physical activity, race/ethnicity

## Abstract

Despite decades of extensive research and clinical insights on the increased risk of all-cause and disease-specific morbidity and mortality due to obesity, the obesity paradox still presents a unique perspective, i.e., having a higher body mass index (BMI) offers a protective effect on adverse health outcomes, particularly in people with known cardiovascular disease (CVD). This protective effect may be due to modifiable factors that influence body weight status and health, including physical activity (PA) and cardiorespiratory fitness (CRF), as well as non-modifiable factors, such as race and/or ethnicity. This article briefly reviews the current knowledge surrounding the obesity paradox, its relationship with PA and CRF, and compelling considerations for race and/or ethnicity concerning the obesity paradox. As such, this review provides recommendations and a call to action for future precision medicine to consider modifiable and non-modifiable factors when preventing and/or treating obesity.

## 1. Introduction

The term obesity paradox refers to the observation that, although being obese is a major risk factor in the development of diseases, such as cardiovascular disease (CVD), adults having obesity coupled with CVD or type 2 diabetes mellitus (T2DM) may have a survival advantage against succumbing to CVD- and T2DM-related health outcomes compared to non-obese adults [1]. Physical activity (PA) is a potent regulator of energy balance (i.e., energy intake relative to energy expenditure) involved in maintaining a healthy weight or losing excess body weight [2, 3], which improves cardiorespiratory fitness (CRF), an important indicator of overall health [4, 5]. As such, in this broad review, we aim to update the evidence on the relationship between PA, CRF, and the obesity paradox. We will also highlight observed racial and/or ethnic differences to inform the clinical application of PA and CRF in healthcare settings to improve patient and clinical management of obesity and its related conditions and diseases.

## 2. The Obesity Epidemic

The obesity epidemic corresponds to the significant and widespread increase in the prevalence of obesity, a medical condition characterized by an excessive accumulation of body fat [6]. This phenomenon has become a global health concern, affecting individuals of all ages, socioeconomic backgrounds, and races and/or ethnicities. Key issues surrounding the obesity epidemic include rising prevalence [7], adverse health consequences [8], a decline in contributing factors related to weight status, including habitual diet intake and quality and PA engagement [9], genetic predisposition [10], environmental (immediate and broad) and societal factors (e.g., cultural norms) [11], economic impact [12], and the intergenerational transmission of obesity [13].

**Fig. 1.**
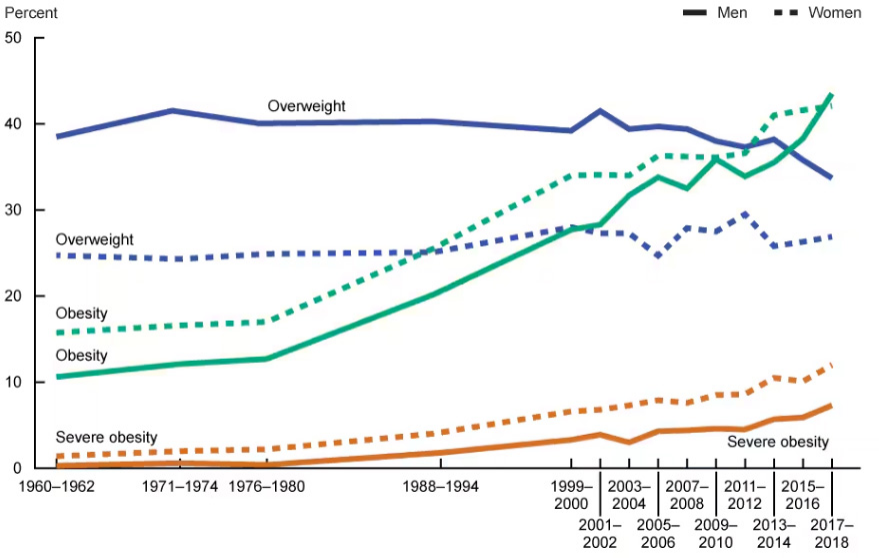
**Trends in overweight or obesity prevalence in the United States of America (included with approval from Fryar *et al*. [14] National Center for Health Statistics Health E-Stats. 2020)**.

Globally, obesity prevalence has increased significantly since the 1960s (Fig. 1, Ref. [14]) [14, 15]. While specific numbers may vary by country and region, in the United States (U.S.), in the 1960s–1970s (13.4–15.0% of the U.S. adult population), obesity rates were relatively low compared to more recently [14]. By the 1980s–1990s (22.9–30.5% of the U.S. adult population), the prevalence of obesity started to rise more prominently. From 2000 to the present, obesity has become a global epidemic (30.5% of the U.S. adult population in 2001–2002 compared to 42.4% of the U.S. adult population in 2017–2018). Many countries have experienced a substantial increase in obesity rates (4.6% of the global adult population in 1980 compared to 14.0% in 2019). This period has been characterized by a growing awareness of the health risks associated with obesity, leading to increased public health efforts to address the issue [16].

Body mass index (BMI) is commonly used as an indicator of obesity (BMI value ≥30 kg/m^2^), as it provides a simple and quick method to assess an individual’s body weight relative to their height [17]. Despite its widespread use, it is important to note that BMI has limitations and does not directly measure body fat or fat distribution or distinguish between total body or site-specific fat mass and fat-free mass.

### Racial and/or Ethnic Differences in Obesity Prevalence

**Fig. 2.**
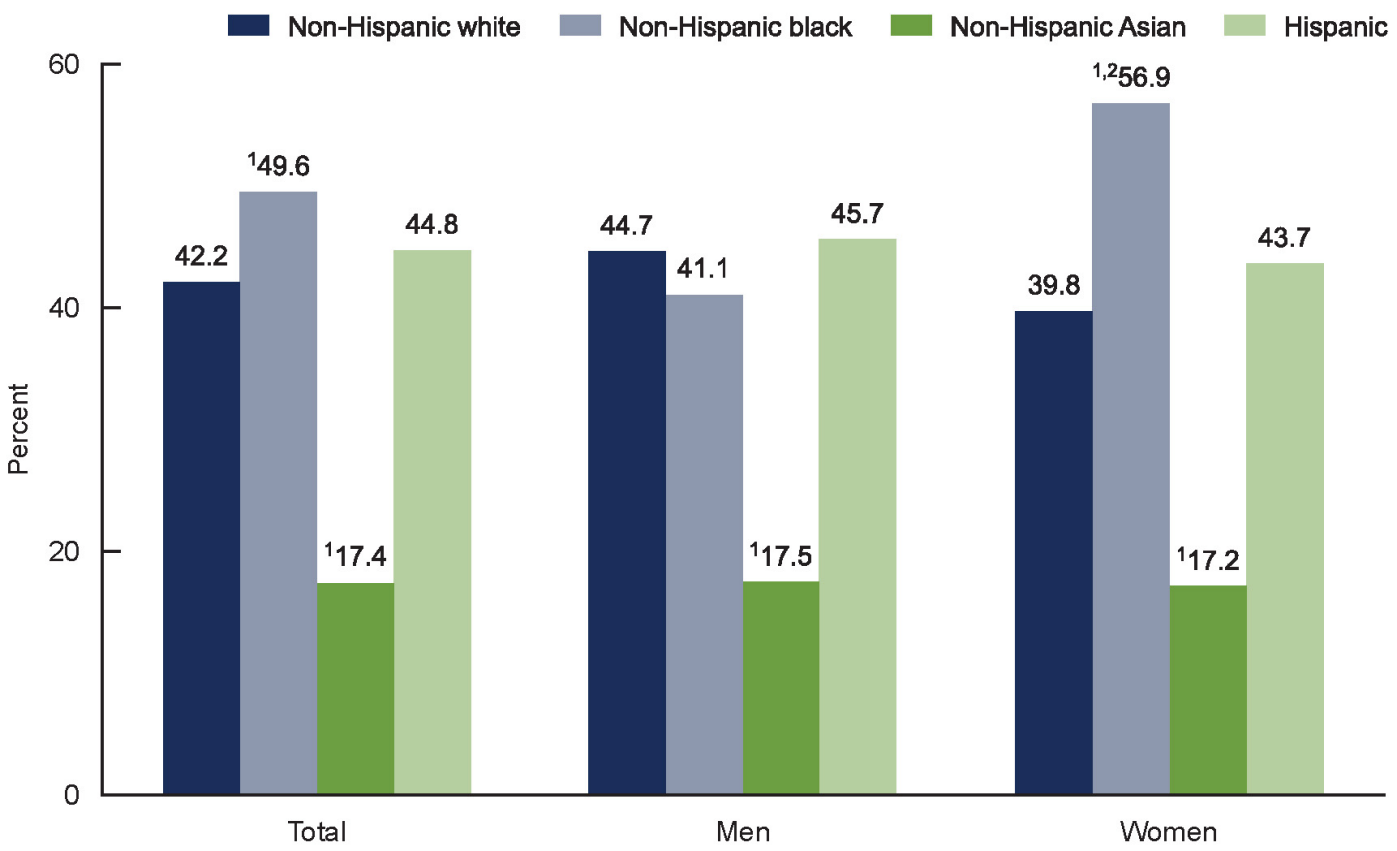
**Prevalence of obesity by race/ethnicity in the United States of America (included with approval from Hales *et al*. [18] Centers for Disease Control and Prevention, National Center for Health Statistics. 2020)**.

There are notable racial and/or ethnical differences in obesity prevalence, and these disparities have been observed in various populations (Fig. 2, Ref. [18]) [18, 19, 20]. It is important to recognize that these differences are influenced by a complex interplay of genetic, environmental, cultural, and socioeconomic factors [21]. In the U.S., obesity rates tend to be higher among Black (African American) and Hispanic populations compared to non-Hispanic white (Caucasian) populations [22]. This pattern is observed across different sexes (i.e., male or female) and/or genders (e.g., transgender) [18, 19, 20] and age groups, including adults and children [23]. Socioeconomic factors, including income and education levels, also affect these disparities, whereby individuals with lower socioeconomic status may face challenges accessing healthy food options and engaging in regular PA. Further, some cultural practices may influence dietary choices [24], and environmental factors, such as urbanization, can potentially impact PA patterns [25]. Evidence suggests that genetic factors may contribute to differences in body composition and metabolism among different racial and/or ethnic groups [26, 27, 28, 29]. However, the role of genetics is complex, while behavioral and environmental factors also play significant roles.

Different racial and/or ethnic populations may exhibit variations in metabolic responses to diet and PA [30]. Thus, understanding these variations is important for developing targeted interventions to improve health outcomes. Racial and/or ethnic disparities in obesity contribute to broader health disparities. As previously stated, obesity is associated with an increased risk of various health conditions, including T2DM, CVD, and certain cancers. Disparities in access to healthcare, preventive services, and health education can also contribute to differences in obesity prevalence and related health outcomes [31]. Addressing racial and/or ethnic disparities in obesity requires a multifaceted approach that considers socioeconomic, cultural, environmental, and individual factors. Strategies should include promoting access to healthy foods, creating supportive environments for PA, culturally tailored health education, and addressing social determinants of health. Moreover, it is essential to approach this issue with sensitivity, recognizing the diverse factors that contribute to obesity disparities and working collaboratively to implement effective interventions that promote health equity.

## 3. The Obesity Paradox

The obesity paradox is a phenomenon observed in certain medical conditions where individuals with a higher BMI, or classified as overweight or mildly obese, may have better health-related outcomes compared to those with a normal or lower BMI [1, 32, 33]. This paradoxical relationship challenges the traditional understanding that obesity is uniformly associated with negative health outcomes [34, 35, 36]. Several health conditions exhibit this paradox, including CVD [37], chronic kidney disease [38], chronic obstructive pulmonary disease [39], certain cancers [40], as well as aging and frailty [41].

Further, individuals who are overweight or have mild obesity (Class 1: 30.0–34.9 kg/m^2^) may have a survival advantage over those with a normal or underweight BMI [42, 43]. Individuals with a higher BMI, especially those with established CVD, have been shown to have better survival rates compared to those with a lower BMI [44]. This paradoxical finding has been observed in other conditions as well, such as heart failure and coronary artery disease [45]. Some studies also suggest that a certain amount of body fat may be protective in older individuals, potentially providing energy reserves during periods of illness or stress [46].

Several hypotheses attempt to explain the obesity paradox, such as survivor bias, metabolic reserve, and underlying health status [47, 48]. It is important to note that the obesity paradox is a complex and debated topic in the medical community [49, 50, 51, 52]. While some studies support the paradox, others emphasize the well-established link between obesity and various health risks, as the relationship between BMI and health outcomes can vary based on factors such as age [46], gender [53], the specific medical condition being studied, and PA and CRF. A critical limitation to the current state of the literature surrounding the obesity paradox is the focus on adults having overweight (25.0–29.9 kg/m^2^) or class 1 obesity. It should be recognized that the general term obesity captures all weight status ≥30.0 kg/m^2^, which potentially has implications on the paradoxical relationship between obesity and non-disease and disease-specific morbidity and mortality. As such, researchers continue to investigate and further our understanding of the obesity paradox and its implications for clinical practice and obesity prevention and treatment.

### Racial and/or Ethnic Considerations for the Obesity Paradox

Although the obesity paradox has been consistently observed across racial and/or ethnic subgroups in large cohorts, some studies have found racial and/or ethnic differences in the strengths of these associations (Table 1, Ref. [54, 55, 56, 57, 58, 59, 60, 61, 62]) [54]. Despite the obesity paradox being observed in several large epidemiologic studies, critics of this phenomenon believe that the detected associations may be related to epidemiological modeling, such as reverse causation, selection bias (survivor bias), competing death risk, and residual confounding [63]. An important confounder might be the utility of BMI as a surrogate for estimating body adipose tissue content, given that BMI does not discriminate between body fluid, adipose tissue, or muscle tissue. However, despite these limitations, analyses in large cohorts examining the relationship between BMI and mortality using several different epidemiological models and accounting for many confounders repeatedly and robustly show similar associations of improved survival for patients with higher BMIs [55, 63, 64, 65]. Yet, differences in these associations across all racial and/or ethnic subgroups have not been fully examined using advanced causal models, and future studies need to address these important considerations. Identifying the unique racial and/or ethnic features of the obesity paradox can better help us understand these mechanisms and/or pathways and provide new risk markers and novel therapeutics to improve survival in these populations.

**Table 1.** **Summary of studies evaluating the impact of race and/or ethnicity on the association between BMI and health-related outcomes (adapted with approval from Kleine *et al*. [54] American Journal of Kidney Diseases. 2018)**.

Author and year	Sample size	Race and/or ethnicity	Study outcomes
Wong *et al*., 1999 [56]	84,192	Asian and Caucasian	In Asian participants, a U-shaped relationship between BMI and mortality risk was noted, with higher mortality observed at the lowest and highest BMI.
Glanton *et al*., 2003 [57]	151,027	African American and Caucasian	BMI ≥30 kg/m^2^ correlated to reduced mortality with a stronger association observed in African American participants.
Johansen *et al*., 2004 [58]	418,055	African American, Asian and Pacific Islanders, Hispanic, and Caucasian	Higher BMI was associated with lower mortality rate in African American, Hispanic, and Caucasian, but not Asian participants.
Ricks *et al*., 2011 [59]	109,605	African American, Caucasian, non-Hispanic, and Hispanic	Higher BMI was associated with survival advantage in African American, Hispanic, and Caucasian participants, with the highest observed in African American participants.
Hall *et al*., 2011 [60]	21,492	Asian, Pacific Islander, Caucasian, and non-Hispanic	Higher BMI was associated with lower mortality in Asian, Pacific Islander, and Caucaisan participants.
Park *et al*., 2013 [61]	40,818	African American, Asian, and Caucasian	Lower mortality risk was observed across higher BMI levels regardless of race and/or ethnicity.
Wang *et al*., 2016 [62]	117,683	African American, Caucasian, non-Hispanic, and Hispanic	Higher BMI was associated with lower mortality risk in African American and non-Hispanic Caucasian participants, while a U-shaped relationship was observed in Hispanic participants, such that lower and higher BMIs were not protective against mortality.
Doshi *et al*., 2016 [55]	123,624	African American, Caucasian, non-Hispanic, and Hispanic	Inverse relationship between BMI and mortality in African American, Hispanic, and non-Hispanic Caucasian participants, with lowest risk for mortality observed in African American participants with a BMI ≥27.5 kg/m^2^.

BMI, body mass index.

## 4. PA, CRF, and the Obesity Paradox

**Fig. 3.**
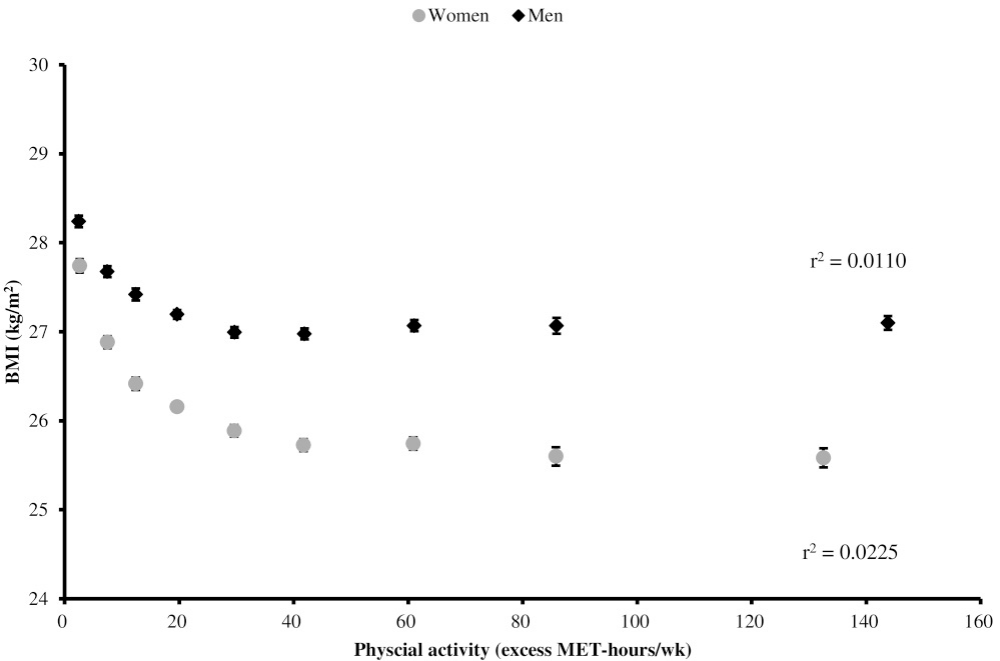
**BMI by levels of PA engagement (included with approval from Bradbury *et al*. [68] BMJ Open. 2017)**. BMI, body mass index; PA, physical activity; MET, metabolic equivalent of task.

PA and BMI are interconnected factors that play crucial roles in determining overall health [33]. PA is defined as any bodily movement produced by skeletal muscles that results in energy expenditure [66]. PA in daily life can be categorized into occupational, sports, conditioning, household, or other activities. Exercise is a subset of PA that is planned, structured, and repetitive and has a final or intermediate objective for improving or maintaining physical fitness. PA may also influence BMI through increased energy expenditure relative to energy intake, skeletal muscle mass relative to fat mass, metabolic health, appetite regulation, and weight maintenance [67]. Previous evidence supports that higher engagement in habitual PA is associated with a lower risk of experiencing overweight or obesity (Fig. 3, Ref. [68]) [68, 69], as well as a decreased risk of all-cause and disease-specific morbidity and mortality [36, 70].

**Fig. 4.**
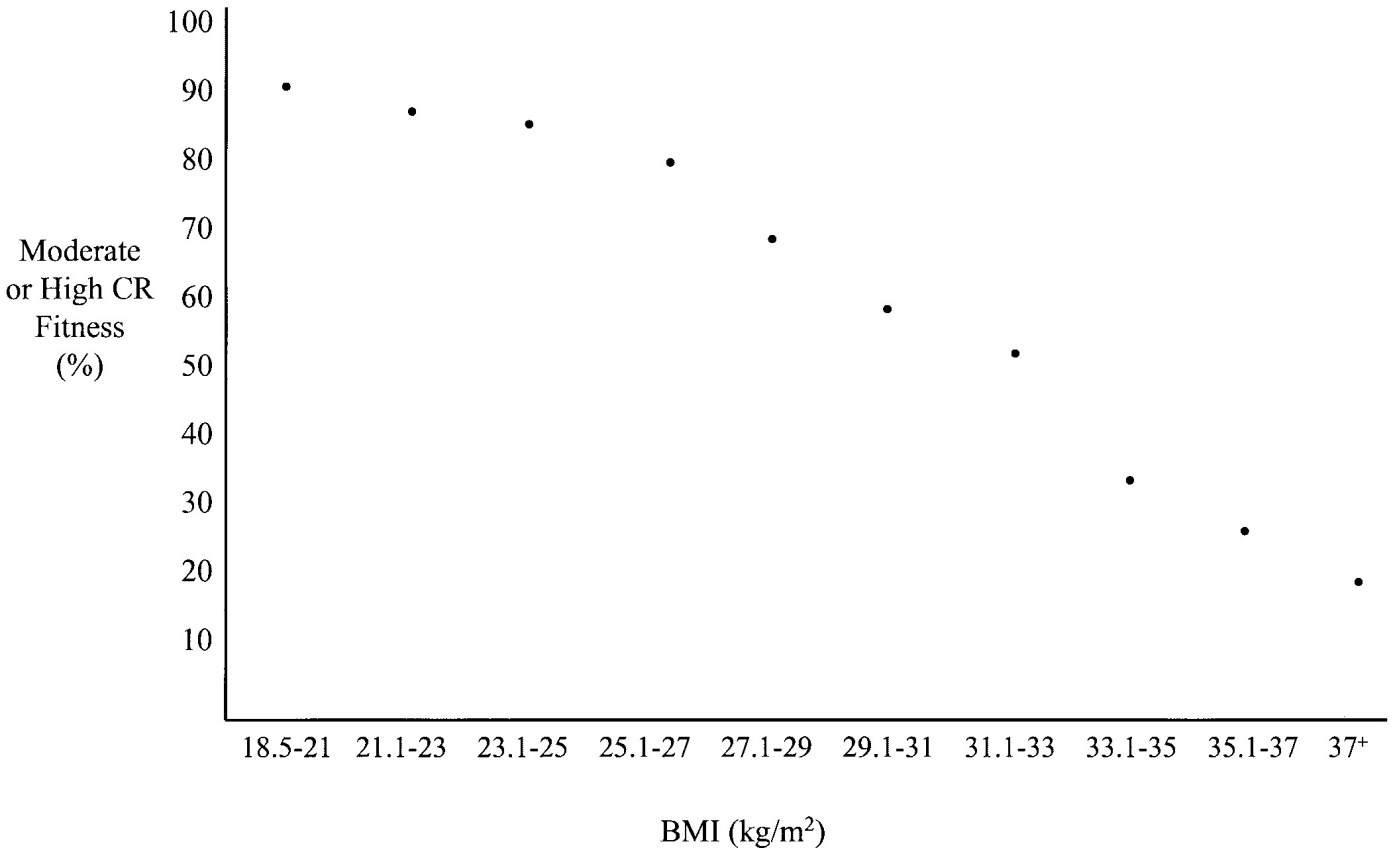
**BMI by CRF level (included with approval from Farrell *et al*. [76] Obesity Research. 2002)**. BMI, body mass index; CRF, cardiorespiratory fitness; CR, cardiorespiratory.

CRF and BMI are both important indicators of overall health and fitness, although they measure different aspects and have distinctly independent implications for health. CRF refers to the ability of the cardiovascular and respiratory systems to supply oxygen to skeletal muscle during sustained PA [71]. CRF is often assessed through measures, such as maximal oxygen consumption (VO_2_ max) or submaximal exercise tests, with a higher VO_2_ max indicating better CRF [72]. Higher CRF is also associated with a reduced risk of CVD, improved metabolic health, and better overall mortality rates [73, 74, 75]. Generally, there is an inverse relationship between CRF and BMI, with higher levels of CRF often associated with a lower BMI (Fig. 4, Ref. [76]) [71, 76, 77, 78], indicating a healthier body weight. Regular aerobic exercise, which improves CRF, can contribute to weight management and obesity prevention [79]. Individuals with better CRF may experience health benefits even if their BMI falls within the overweight or obese categories [80]. Collectively, CRF and BMI are complementary measures that, when considered together, provide a more comprehensive picture of an individual’s health. In summary, while BMI provides a useful screening tool for weight status, CRF offers insights into cardiovascular and respiratory health and overall fitness.

Over the past 20+ years, the “fat but fit” concept has supported the obesity paradox through the lens of PA engagement and CRF improvement [81]. The idea of “fat but fit” refers to those individuals who, despite having obesity, have a relatively high CRF [82]. Two seminal manuscripts utilizing data from the Aerobics Center Longitudinal Study (ACLS) supported this and are considered the foundation of the “fat but fit” concept [83, 84]. These studies demonstrated that all-cause and CVD-specific mortality risks in individuals having obesity, defined by BMI, body fat percentage, or waist circumference, with CRF levels above the age-specific and sex-specific 20th percentile, were not significantly different from their normal weight and fit counterparts, which would theoretically be considered the healthiest possible group. Therefore, when targeting risk reductions in all-cause and disease-specific morbidity and mortality associated with obesity, both weight and/or fat reduction and CRF improvement should be considered.

### 4.1 Racial and/or Ethnic Differences in PA and CRF

**Fig. 5.**
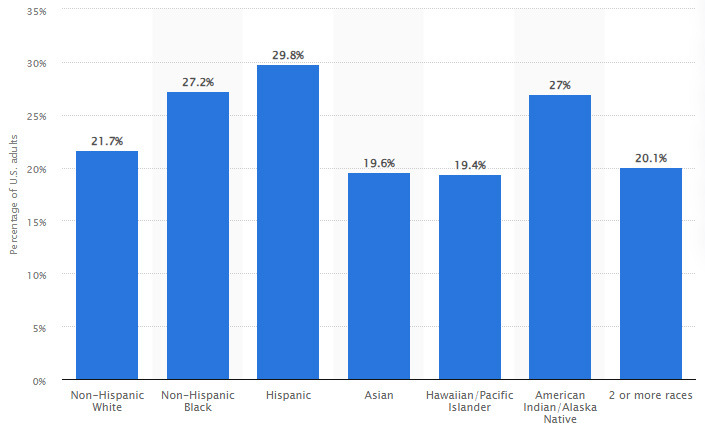
**Prevalence of physical inactivity by race and/or ethnicity (included from Statista)**.

PA participation can vary among racial and/or ethnic groups (Fig. 5) [85, 86, 87, 88]. Cultural values, traditions, and practices can significantly influence PA behaviors [89]. Social norms within a particular community may affect perceptions of PA and exercise [90]. The availability of safe and accessible spaces for PA, such as parks, sidewalks, and recreational facilities, varies across local, regional, national, and international locations [91, 92]. Some communities may face challenges related to the built environment, impacting opportunities for PA. Socioeconomic factors, including income and education levels, affect PA patterns. Individuals with lower socioeconomic status may have limited access to resources such as gym memberships, organized sports, or recreational facilities. The nature of occupations within different racial and/or ethnic groups may also influence overall PA levels, such that occupations that involve physical labor may contribute to higher levels of occupational PA [93].

**Fig. 6.**
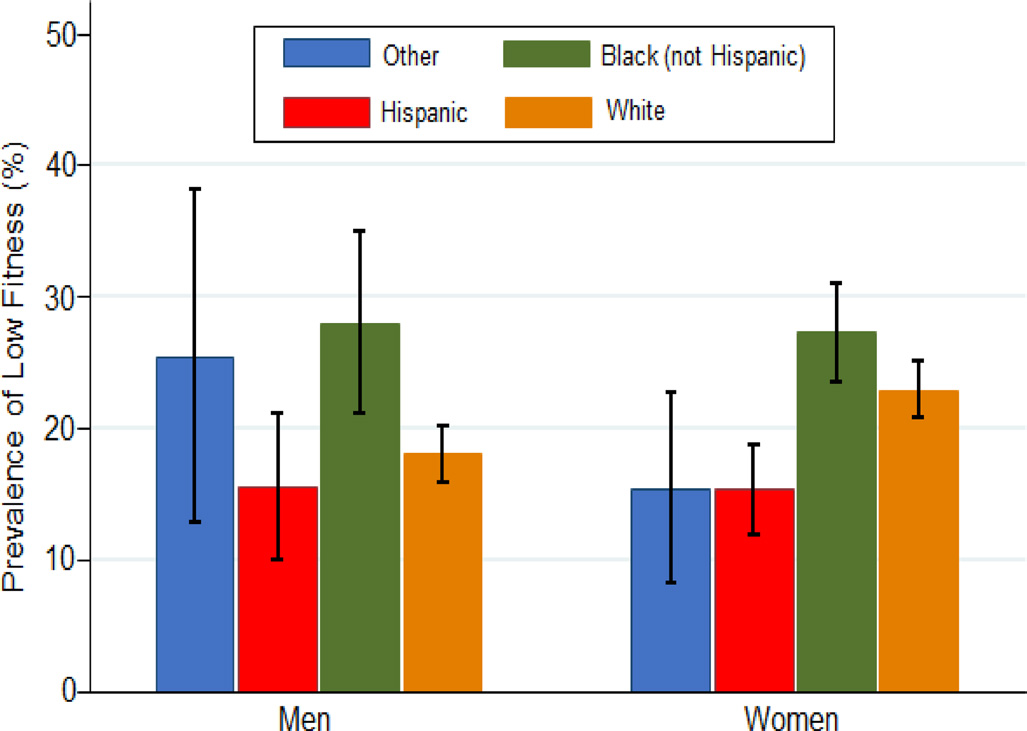
**Prevalence of low CRF by race/ethnicity (included with approval from Kaze *et al*. [94] BMJ Open. 2022)**. CRF, cardiorespiratory fitness.

Similar to PA, CRF can vary among different racial and/or ethnic groups (Fig. 6, Ref. [94]) [94, 95, 96, 97]. Evidence suggests that genetic factors may contribute to individual differences in CRF. Some studies indicate that certain genetic variations may influence an individual’s response to aerobic exercise and impact their overall CRF [98, 99, 100, 101]. Ethnicity is a complex concept that includes genetic, cultural, and social dimensions [102, 103]. Within any racial or ethnic group, there is substantial genetic diversity, and genetic influences on CRF may vary among individuals [101, 104]. Cultural values, traditions, and lifestyle preferences can influence PA patterns and CRF levels [89]. Some cultural practices may include PA as an integral part of daily life, while others may not emphasize structured PA. Dietary habits, influenced by cultural factors, can also play a role in CRF, as nutrition contributes significantly to overall health and fitness [105]. Certain health conditions can affect an individual’s ability to exercise regularly and influence their overall fitness, and health disparities among different racial and ethnic groups, including the prevalence of chronic conditions, may impact CRF.

### 4.2 Racial and/or Ethnic Differences in the Relationship between PA, CRF, and Obesity

The relationship between PA, CRF, and obesity varies among different racial and/or ethnic groups [106, 107]. Genetic factors may contribute to individual variability in metabolism, fat distribution, and the physiological response to exercise [108, 109, 110, 111, 112]. Cultural values, traditions, and lifestyle preferences influence dietary habits, PA patterns, and overall health behaviors, which may contribute to variations in the relationship between PA, CRF, and obesity. Cultural norms regarding body image and beauty standards may influence attitudes toward PA and obesity within different communities [113, 114]. As such, racial and/or ethnic disparities in the prevalence of chronic conditions (e.g., T2DM, CVD) may bidirectionally influence body weight, PA levels, and CRF. It has previously been demonstrated that lower levels of CRF exist among African Americans compared to Caucasians [115], for example, and observed that African Americans also appear to have fewer improvements in CRF following formal exercise training programs [116]. These data suggest that special efforts are needed to improve body composition metrics in African Americans and other high-risk and/or minoritized groups and progress levels of PA directed at enhancing levels of CRF [117].

## 5. Conclusions, Public Health Implications, and a Call to Action

Obesity is a public health crisis that disproportionately affects vulnerable populations, specifically racial and/or ethnic minorities [118]. This disparity is rooted in a complex interplay of social, economic, and cultural factors [119]. Racial and ethnic disparities in health outcomes may stem from systematic differences in PA engagement and CRF between minority populations and the majority. Various factors contribute to these disparities, including limited access to safe and conducive environments for exercise, disparities in recreational facilities, and cultural preferences influencing activity patterns. Systematic differences in CRF levels may arise due to these disparities, impacting cardiovascular health and overall well-being.

The cumulative effect of these factors amplifies the obesity burden among racial and/or ethnic minorities. Thus, recognizing these inequalities is crucial for promoting health equity. Interventions that address environmental barriers, foster inclusive PA programs and consider cultural contexts can contribute to narrowing the gap in PA engagement and CRF improvement, ultimately enhancing health outcomes in racial and/or ethnic minorities. Addressing this necessitates a comprehensive approach to socioeconomic inequalities and cultural competence in healthcare and the implementation of policies fostering equitable access to healthy living resources.

Precision approaches to prescribing PA represent a warranted and promising strategy for enhancing CRF [120]. By recognizing the individual variability in responses to PA and exercise, these tailored approaches consider factors such as genetics, current fitness levels, health status, and personal preferences. Moreover, by leveraging precision medicine principles, healthcare professionals can design personalized exercise regimens that optimize CRF while minimizing potential risks. This targeted approach enhances the effectiveness of PA interventions and fosters long-term adherence by aligning with individual capabilities and preferences [121]. Embracing precision approaches in prescribing PA not only holds the potential to advance CRF outcomes but also reflects a commitment to personalized, patient-centered healthcare that acknowledges and addresses the unique needs of each individual.

A compelling call to action is imperative to drive policy-level changes to improve the broader environment and promote public health. By recognizing the influential role of policy in shaping societal norms and behaviors, an urgent need arises for initiatives that advocate for healthier environments, equitable access to resources, and the dismantling of systemic barriers [119]. Policymakers should prioritize interventions that address social determinants of health, such as affordable access to nutritious foods, safe recreational spaces, and healthcare services. Additionally, comprehensive policies can combat food deserts, incentivize businesses to promote health-conscious practices, and establish standards for urban planning that prioritize walkability and PA. A call to action should mobilize diverse stakeholders, including government bodies, community leaders, and advocacy groups, to collaborate on evidence-based policy solutions. By galvanizing support for systemic change, we can foster environments that facilitate healthier choices, reduce health disparities, and cultivate a culture of well-being for all. An overarching call to action as it relates to the obesity paradox is to include a holistic approach to routine preventive care, which accounts for weight status in addition to other modifiable (PA and CRF) and non-modifiable (race and/or ethnicity) risk factors that may impact overall and disease-specific morbidity and mortality. If clinicians and practitioners merely treat obesity as a chronic disease without consideration for other risk factors, the obesity paradox may not be accounted for in routine preventive care.

Lastly, a call to action dedicated to clinicians and practitioners is to make educated recommendations for lifestyle modifications in the context of the obesity paradox. In addition to understanding the social determinants of health (e.g., race and/or ethnicity) and how they may impact PA, CRF, and the obesity paradox, practitioners should utilize and integrate other health-based assessments to determine appropriate lifestyle modification. Within recent years, physical activity has been increasingly recognized as a vital sign of overall health, alongside traditional metrics such as weight status (e.g., obesity), blood pressure (e.g., hypertension), cholesterol (e.g., hypercholesterolemia), and glucose (e.g., T2DM) [122]. Further, the Exercise is Medicine (EIM) initiative is a global health campaign launched by the American College of Sports Medicine (ACSM) aimed at promoting physical activity as a vital component of preventing and managing chronic diseases [123, 124]. EIM advocates for integrating exercise assessment and physical activity prescription into routine healthcare practices, aiming to improve public health and quality of life [125]. In addition to measuring and assessing CRF through exercise testing, the incorporation of non-exercise estimated CRF (eCRF) has emerged as a valuable tool for determining cardiovascular health and risk stratification, offering several benefits for inclusion in medical practice [126, 127]. Non-exercise eCRF methods, including prediction equations and/or algorithms based on demographic, anthropometric, and lifestyle factors, provide a convenient and cost-effective way to estimate an individual’s CRF without needing exercise testing [128, 129, 130]. Collectively, the clinician and practitioner have a unique set of tools for treating obesity and its paradox.

## Abbreviations

ACLS, Aerobics Center Longitudinal Study; ACSM; American College of Sports Medicine; BMI, body mass index; CRF, cardiorespiratory fitness; CVD, cardiovascular disease; eCRF, estimated cardiorespiratory fitness; EIM, Exercise is Medicine; PA, physical activity; T2DM, type 2 diabetes mellitus; VO_2_ max, maximal oxygen consumption.
